# Combination of B-cell-guided rituximab and low-dose tacrolimus for primary membranous nephropathy: a retrospective cohort study

**DOI:** 10.3389/fmed.2026.1761271

**Published:** 2026-03-10

**Authors:** Lijiao Wang, Shuai Huo, Zhenzhen You, Yang Dong, Yuan Gan, Zhu Zhang, Yue Gu, Lei Yan, Fengmin Shao

**Affiliations:** 1 Department of Nephrology, Fuwai Central China Cardiovascular Hospital, Central China Fuwai Hospital of Zhengzhou University, Zhengzhou, China; 2 Department of Pharmacy, Fuwai Central China Cardiovascular Hospital, Central China Fuwai Hospital of Zhengzhou University, Zhengzhou, China; 3 Department of Nephrology, Henan Provincial Key Laboratory of Nephrology and Immunology, Henan Provincial Clinical Research Center for Nephrology, Henan Provincial People’s Hospital, Zhengzhou, China

**Keywords:** B-cell-guided, B-cell-targeted therapy, primary membranous nephropathy, rituximab, tacrolimus

## Abstract

**Background:**

B-cell-targeted therapy with rituximab (RTX) has become a first-line option for primary membranous nephropathy (PMN), but current regimens incur substantial medical costs, and some patients show suboptimal responses. The combination of RTX with immunosuppressants has garnered increasing interest, yet its efficacy and optimal dosing remain unclear. This study compared the efficacy and safety of B-cell-guided RTX combined with low-dose tacrolimus (TAC) versus standard RTX monotherapy for the treatment of PMN.

**Methods:**

This retrospective analysis included 116 patients diagnosed with PMN between December 2022 and December 2024. All participants were diagnosed based on clinical evaluation and renal biopsy pathology. Of these patients, 57 finally received B-cell-guided RTX combined with low-dose TAC (observation group), while 38 received RTX monotherapy (standard group). In the observation group, RTX dosage was adjusted based on B-lymphocyte counts until B-cell depletion was achieved, accompanied by long-term oral low-dose TAC (0.02 mg/kg/day). Follow-up was conducted monthly after treatment to monitor peripheral blood lymphocyte subsets. RTX was re-administered if CD19+ B-lymphocytes rebounded to >5 cells/μL and the patient had not achieved complete remission. The standard group received two 1 g doses of administered 2 weeks apart. Over a 6-months follow-up, remission rates, incidence of adverse events, and treatment costs were compared between the two regimens.

**Results:**

The overall remission rate in the observation group was 71.93%, with complete and partial remission rates of 31.58% and 40.35%, respectively. In the standard group, the overall remission rate was 68.42%, with complete and partial remission rates of 26.32% and 42.1%, respectively; none of these differences were statistically significant (*P* > 0.05). Multi-factor Logistic regression analysis identified non-use of renin-angiotensin system inhibitors (RASi) as an independent risk factor for non-remission (OR = 9.113, 95% CI: 1.010, 82.259, *P* = 0.049), while higher baseline albumin levels served as a protective factor for remission (OR = 0.862, 95% CI: 0.747, 0.995, *P* = 0.042). The observation group received a significantly lower total dose of RTX (0.3 ± 0.16g vs. 2 g, *t* = 73.19, *P* = 0.000) and had reduced immunosuppressive therapy costs (5608.77 ± 2053.41 CNY vs. 26,000 CNY, *t* = 74.973, *P* = 0.000), resulting in average savings of approximately 20,391.23 CNY per patient. During treatment, no serious adverse events occurred in the observation group, whereas four serious adverse events were reported in the standard group. Non-serious adverse events totaled 12 in the observation group and 18 in the standard group. Overall safety was significantly higher in the observation group (χ^2^ = 12.656, *P* = 0.001).

**Conclusion:**

B-cell-guided RTX combined with low-dose TAC effectively induces clinical remission in patients with PMN, with a lower total RTX dose, improved safety profile, and better cost-effectiveness.

## Introduction

Primary membranous nephropathy (PMN) is one of the most common causes of nephrotic syndrome in adults, accounting for 23.4% of all renal biopsy in China (1). The prognosis of PMN varies significantly. Approximately 30% of patients can achieve spontaneous remission, while 30% of patients fail to respond to immunosuppressive therapy and progress to end-stage renal disease (ESRD) (2–4). Persistent heavy proteinuria is an important risk factor for renal function deterioration (5). Therefore, the KDIGO 2021 Clinical Practice Guideline for the Management of Glomerular Diseases recommends individualized management of PMN based on the risk of progressive renal function decline (6). Although traditional immunosuppressive regimens, such as combinations of glucocorticoids (GC) with alkylating agents or GC with calcineurin inhibitors (CNIs)–are effective, they carry risks including infection, bone marrow suppression, nephrotoxicity, and a high recurrence rate (7).

In recent years, with the successive identification of podocyte target antigens such as M-type phospholipase A2 receptor (PLA2R) and thrombospondin type 1 domain-containing 7A (THSD7A) (8, 9), PMN is recognized as an organ-specific autoimmune podocytopathy (10–12). B lymphocytes generate antibodies that bind to podocyte antigens, activate the complement system (13), trigger oxidative stress and inflammation (14), and damage the podocyte filtration barrier. Environmental pollutants (such as PM2.5) can promote oxidative stress, and RAS signaling via the Wnt1/β-catenin pathway can further worsen podocyte injury (15, 16).

Given the central pathogenic role of B lymphocytes in PMN (17), B cell–targeted therapy with rituximab (RTX) can selectively deplete B cells and suppress production of pathogenic antibodies, and it has become a first-line immunosuppressive option for high-risk PMN patients (18–21). Current RTX regimens mirror those used in lymphoma, employing either a “two-dose” or a “four-dose” schedule. However, in PMN patients, both the number and activity of B cells are substantially lower than that in lymphoma patients, leading to ongoing debate about the need for equivalent dosing (22). Moreover, 20%–40% of patients fail to respond to the initial course of RTX, and 5%–28% relapse after remission (20, 21). CNIs, such as tacrolimus (TAC) and cyclosporine, are less effective at eliminating autoantibodies but mitigate immune-mediated injury by inhibiting T-lymphocyte activation and proliferation (23). TAC is associated with a high risk of disease relapse following discontinuation and carries a significant potential for nephrotoxicity with prolonged use. Consequently, combination therapy using RTX and CNIs has attracted interest (24). Recent studies report that treating PMN with RTX and cyclosporine (CsA) results in higher remission rates and faster remission compared to RTX monotherapy, along with stable renal function and good tolerability (25, 26). In addition, the high price of RTX increases the economic cost pressure on patients’ treatment. Our study attempts to explore an economical and effective treatment regimen by comparing combination therapy using B-cell-guided RTX and low-dose TAC to the standard two-dose RTX protocol.

## Materials and methods

### Study participants

This study is a retrospective cohort study. Patients with PMN who were treated in the Department of Nephrology of Fuwai Central China Cardiovascular Hospital from December 2022 to December 2024 were included. The enrollment criteria were as follows: (1) age ≥ 18 years; (2) PMN was diagnosed by renal biopsy, including newly-treated patients at risk of disease progression and re-treated patients who did not achieve remission or relapsed after treatment with glucocorticoids/other immunosuppressive agents. (3) Estimated glomerular filtration rate (eGFR) > 30 ml/min/1.73 m^2^. The exclusion criteria were as follows: (1) patients with active infections, or in the active stage of hepatitis B or C; (2) pregnancy; (3) B-lymphocyte tumors or other presence of tumors. This study was approved by the Ethics Committee of Fuwai Central China Cardiovascular Hospital.

### Study design

The enrolled patients were divided into two groups according to different RTX regimens: (1) B-cell-guided RTX combined with low does TAC was defined as the observation group: a single intravenous infusion of 0.1 g RTX [Hanlikang, Shanghai Fosun Pharmaceutical (Group) Co., Ltd.,] was administered. If B-cell depletion is not achieved, additional RTX are administered until depletion is confirmed. While low dose TAC (Saifukai, Hangzhou Zhongmei Huadong Pharmaceutical Co., Ltd.) [0.02 mg/(kg⋅d)] was taken orally for long-term maintenance. The peripheral blood CD19+ B cells are monitored monthly. If B-cell reconstitution occurs and the patient did not achieve complete remission, RTX treatment was repeated. (2) The standard two-dose RTX regimen with 1 g RTX, followed by a repeated dose 2 weeks later was defined as the standard group. Before RTX infusion, all patients in both groups received standard premedication to prevent acute infusion reactions. This included infusion reactions. Electrocardiographic monitoring was performed throughout the infusion process to detect changes in heart rate, blood pressure, and oxygen saturation. All patients received compound sulfamethoxazole to prevent *Pneumocystis jirovecii* pneumonia during the treatment period.

### Monitoring indicators

The clinical and biological data of patients were recorded. Clinical data included age, gender, height, weight, renal pathological staging, and disease duration. Biological indicators comprised serum PLA2R antibody(anti-PLA2R), serum albumin (Alb), serum creatinine (SCr), serum cystatin C (Cys C), total serum cholesterol (TC), triglyceride (TG), hemoglobin (Hb), serum C-reactive protein (CRP), D-dimer (Dimer), 24-hour urine total protein (24h-UTP), and lymphocyte subset counts, which were monitored at baseline and 1, 3, and 6 months of follow-up. Adverse reactions were also recorded simultaneously.

### Criteria for efficacy evaluation

B-cell depletion (BCD): peripheral blood CD19+ B cell counts < 5 cells/ul.

B-cell reconstitution: peripheral blood CD19+ B cell counts greater than 5 cells/ul after BCD has been achieved.

Complete remission (CR): 24h-UTP < 0.5 g.

Partial remission (PR): 24h-UTP ≥ 0.5 g but <3.5 g, or as a ≥50% reduction in 24h-UTP from baseline with stable renal function (serum creatinine rise < 20% from baseline).

Cases not meeting CR or PR were considered invalid.

### Statistical analysis

Statistical analysis and chart drawing were performed using Statistical Product and Service Solutions (SPSS) 25.0 (IBM Corp., NY, USA) and GraphPad Prism 8.0 software. Continuous variables with normal distribution were expressed as mean ± standard deviation (x ± s), those with non-normal distribution were expressed as median and interquartile range (M, IQR), and categorical variables were expressed as frequencies or percentages. *T*-test or Mann-Whitney U test was used for the comparison between groups of continuous variables, and analysis of variance was used for repeated-measurement comparison, with the Bonferroni method used to control type I errors. The chi-square test or Fisher’s exact test was used to determine differences between categorical variables. Logistic regression was used to control confounding factors and identify non-remission risk factors. Statistical significance was set at *P* < 0.05.

## Results

### Baseline data status

**FIGURE 1:**
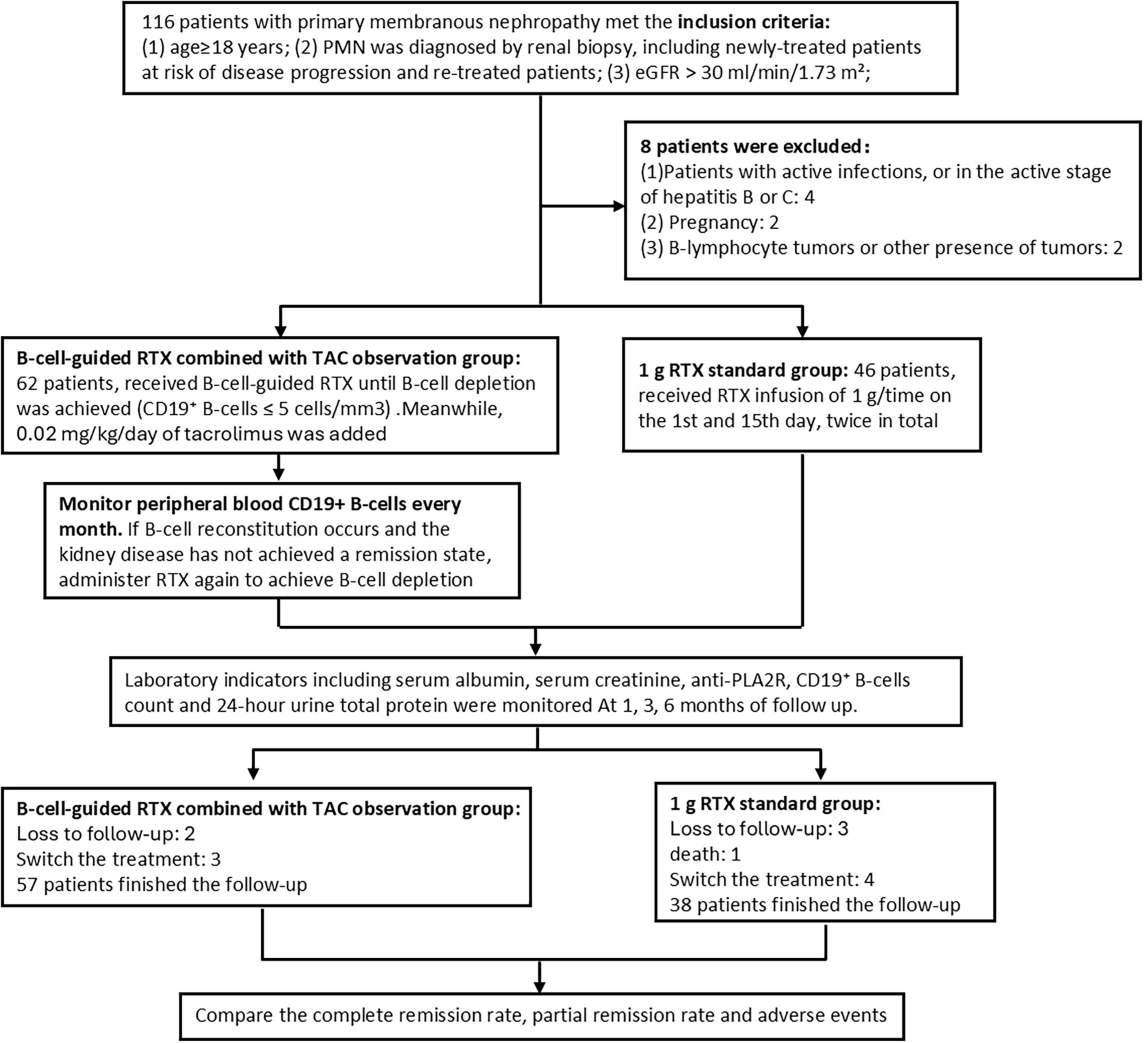
Flow chart of the study. The flowchart shows the patient selection process in this retrospective cohort study. The cohort included 116 patients with PMN between December 2022 and December 2024. After screening under the conditions shown in this Figure, 95 eligible patients (the observation group, *n* = 57; the standard group, *n* = 38) were included in the final analysis.

A total of 116 patients with PMN were enrolled. During the follow-up process, eight patients were excluded for reasons such as active infections, pregnancy and tumors. The observation group and standard group included 62 and 46 patients, respectively. Five patients were lost to follow-up and seven patients switched the treatment in mid-treatment, while one patient died of leukoencephalopathy in the standard group. Finally, 95 patients completed the follow-up study, the observation group included 57 patients, while the standard group included 38 patients (Figure 1).

No statistically significant differences were found between the two groups in clinical data at baseline including age, gender, BSA, serum albumin, total cholesterol, serum creatinine, serum cystatin C, hemoglobin, 24-hour urine protein, anti-PLA2R (*P* > 0.05). There were also no statistical differences in the proportions of lymphocyte subsets between the two groups, including the number of CD19+ B lymphocytes, the CD4/CD8 ratio, and the number of NK cells. Type I membranous nephropathy was the main pathological staging in both groups. Renin-angiotensin system inhibitors (RASi) were recommended for all patients unless contraindicated or intolerant, and there was no significant difference in the usage rate of concomitant medications between the two groups. The proportion of relapse treatment in the observation group was higher (χ^2^ = 5.309, *P* = 0.027), and the disease duration was longer [24 (9,60) vs. 3 (1,9), *Z* = −4.578, *P* = 0.000] than that in the standard group. The difference between the two groups was statistically significant (Table 1).

**TABLE 1:** Comparison of baseline data between the two groups.

Clinical characteristics	Standard group (*n* = 38)	Observation group (*n* = 57)	t/Z/χ^2^	*P*-value
Age (years)	51.58 ± 11.512	49.60 ± 12.967	0.674	0.502*
Male *n* (%)	27 (71.05%)	38 (66.67%)	0.203	0.822&
Body surface area (m^2^)	1.76 ± 0.15	1.75 ± 0.18	0.371	0.712*
Relapse treatment *n* (%)	20 (52.63%)	43 (75.43%)	5.309	0.027&
Disease duration (month)	3 (1,9)	24 (9,60)	−4.578	0.000#
**Pathological stage *n* (%)**				
I	19 (50%)	29 (50.88%)	0.926	0.629&
II	17 (44.74%)	27 (47.37%)		
III	2 (5.26%)	1 (1.75%)		
**Concomitant drugs *n* (%)**				
RASi	36 (94.73%)	50 (87.71%)	1.309	0.253&
Serum albumin (g/L)	24.63 ± 6.68	27.75 ± 9.35	−1.893	0.062*
Total cholesterol (mmol/L)	7.22 ± 2.22	6.78 ± 2.61	0.845	0.401*
Triglyceride (mmol/L)	3.39 ± 2.69	2.46 ± 1.46	2.173	0.032*
Serum creatinine (umol/L)	78.73 ± 30.02	95.61 ± 57.21	−1.874	0.064*
Serum cystatin C (mg/L)	1.01 (0.88, 1.08)	0.99 (0.76, 1.51)	−0.053	0.958#
Hemoglobin (g/L)	131.24 ± 18.75	124.04 ± 19.50	−1.796	0.077*
24-hour urine protein (g)	8.15 ± 5.68	7.77 ± 5.47	0.334	0.739*
Anti-PLA2R	71.45 (17.91, 267.07)	70.09 (12.77, 230.16)	−0.057	0.955#
Number of CD19+ B-cells count (/ul)	302.25 ± 174.96	266.71 ± 189.09	0.924	0.358*
CD4/CD8	2.16 ± 1.66	1.94 ± 0.82	0.719	0.476*
Number of NK lymphocytes (/ul)	207.41 ± 132.14	208.98 ± 135.59	−0.054	0.957*

*Continuous variables with normal distribution were expressed as (x ± s), the group comparisons were used the “*T*- test”. #Non-normal distributed continuous were expressed as (M, IQR), the group comparisons were used the “Mann-Whitney U test”. &Categorical variables were expressed as frequencies or percentages, the group comparisons were analyzed with the “Chi-square test” or “Fisher’s exact test” (as appropriate).

### Disease remission status

**FIGURE 2:**
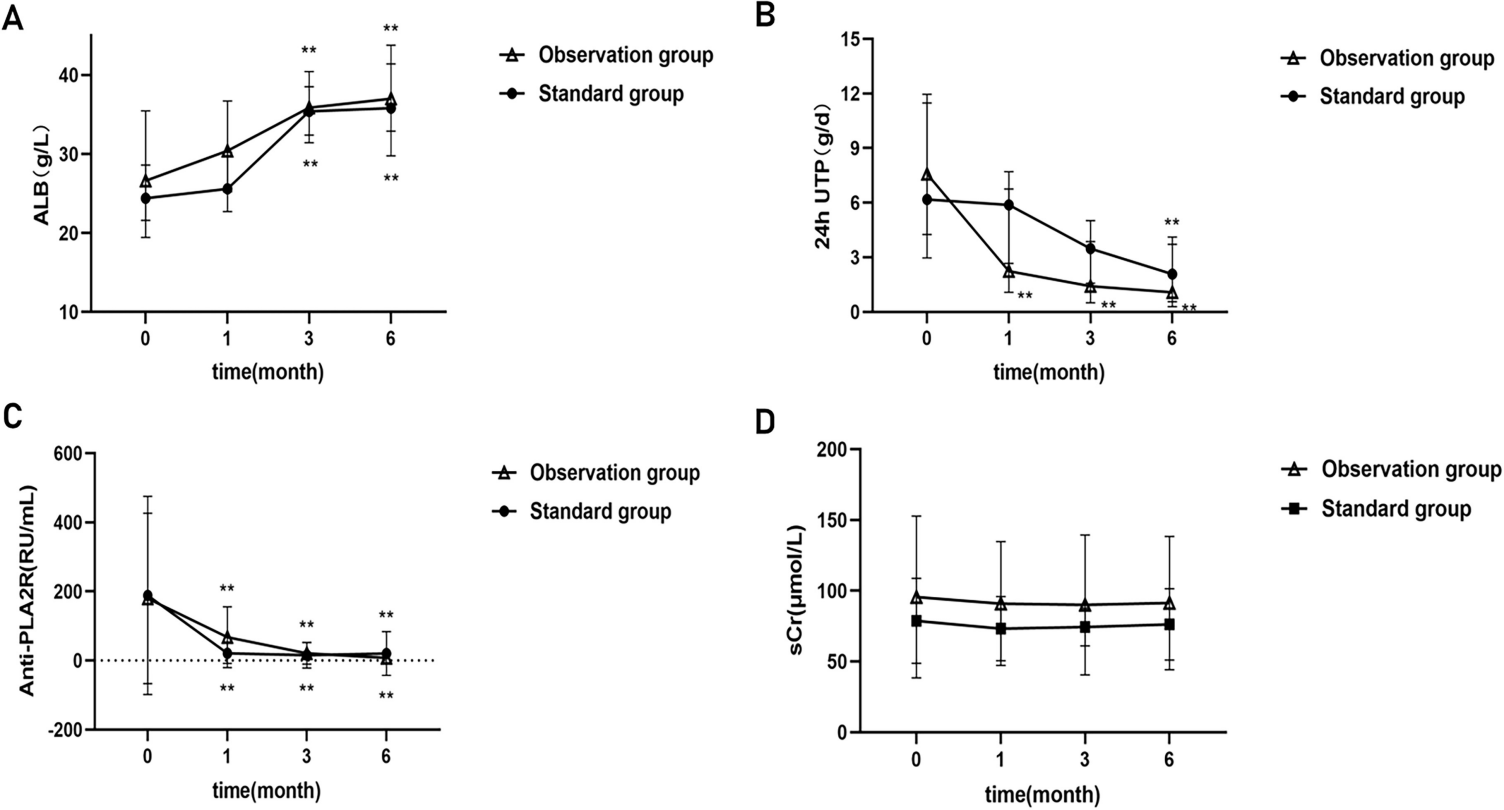
Comparison of the clinical data between two groups at baseline and follow-up. **(A)** Increase in serum albumin. **(B)** Decrease in 24h-UTP; **(C)** decrease in anti-PLA2R. **(D)** Serum creatinine remains stable. *P < 0.05, **P < 0.01, *P* < 0.05 indicates significant difference.

The median follow-up time was 11 months (interquartile range 6–24 months). The results indicated that during the follow-up period, the patient’s 24-hour urinary total protein level progressively decreased, accompanied by a gradual increase in albumin. The anti-PLA2R titer showed a declining trend, and the serum creatinine level remained largely stable. At 1 month of treatment, the serum albumin level in the observation group was significantly higher than that in the standard group (30.48 ± 7.67 vs. 26.53 ± 6.95, *t* = −2.398, *P* = 0.019), and the same with the anti-PLA2R titer [19.56 (4.43, 100.45) vs. 9.41 (1.00, 23.02), *Z* = −2.502, *P* = 0.012], a statistical difference was noted between the two groups. However, no significant difference was noted in 24-hour urinary total protein levels. At 6 months, compared to baseline, both two groups showed significant decreases in 24-hour urinary total protein significantly, significant increases in serum albumin, and significant reductions in anti-PLA2R antibody titers, but no statistically significant differences were observed between the two groups (Figure 2).

**FIGURE 3:**
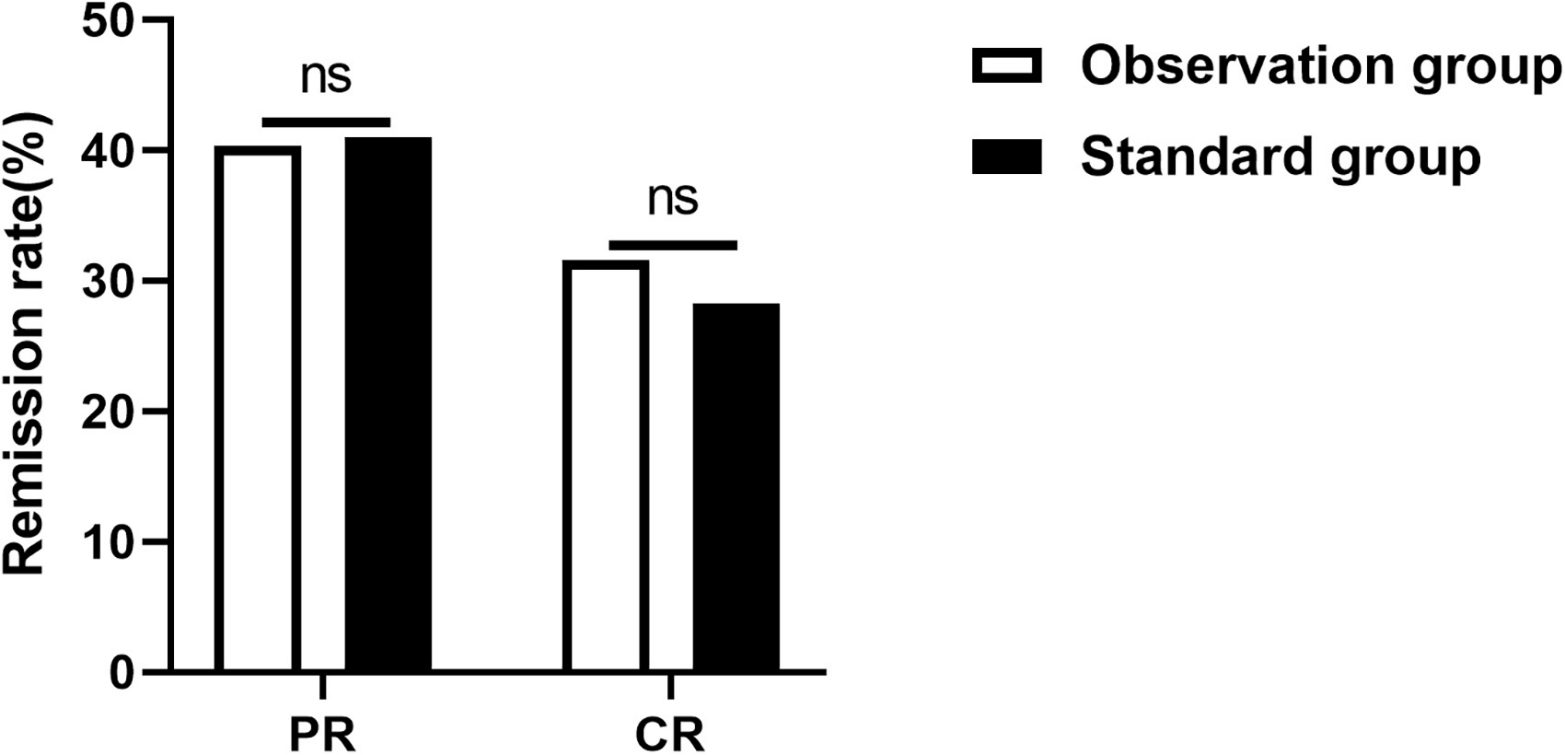
Comparison of the 6-month remission rates between the two groups. ns, no significant difference, *P < 0.05, **P < 0.01, *P* < 0.05 indicates significant difference.

In our study, the overall remission rate of the observation group was 71.93% at the 6-months follow-up, compared with 68.42% in the RTX standard group (χ^2^ = 0.082, *P* = 0.475), no statistically significant differences were observed between the two groups. In detail, the complete and partial remission rates of the observation group were 31.58% and 40.35%, respectively. Meanwhile, those of the standard group were 26.32% and 42.1%, respectively. There were no statistically significant differences between the two groups (χ^2^ = 0.327, *P* = 0.849) (Figure 3).

Multi-factor Logistic regression analysis was used to identify the risk factors for non-remission of PMN after 6 months of treatment, with baseline variables incorporated via forced entry method for equation modeling. The findings revealed that: Non-use of RASi (odds ratio [OR] = 9.113, 95% confidence interval [CI] 1.010, 82.259, *P* = 0.049) was an independent risk factor for non-remission of PMN at 6 months post-treatment. Meanwhile, higher baseline albumin levels (OR = 0.862, 95% CI 0.747, 0.995, *P* = 0.042) served as a protective factor for disease remission. In addition, treatment regimens did not exert a statistically significant impact on remission outcomes (OR = 0.904, 95% CI 0.242, 3.381, *P* = 0.881) (Table 2).

**TABLE 2:** Non-remission risk factors in patients with PMN.

Clinical characteristics	β	*P*	OR	95% CI
Previous treatment (relapse/initial treatment)	0.164	0.810	1.179	0.309, 4.491
Disease duration (month)	0.000	0.984	1.000	0.983, 1.018
Combined use of RASi (none-used/used)	2.210	0.049*	9.113	1.010, 82.259
Serum albumin (g/L)	−0.148	0.042*	0.862	0.747, 0.995
Triglyceride (mmol/L)	−0.142	0.459	0.868	0.596, 1.264
24-hour urine protein (g)	−0.080	0.308	0.923	0.791, 1.077
Anti-PLA2R (titer)	−0.016	0.451	0.984	0.943, 1.026
Serum creatinine (umol/L)	0.011	0.447	1.011	0.983, 1.040
Different treatment groups (the observation group/the standard group)	−0.101	0.881	0.904	0.242, 3.381

Logistic forced inclusion analysis, *n* = 95. *P < 0.05 indicates significant difference.

### B-cell depletion status

During the follow-up period, lymphocyte subsets were monitored continuously to evaluate the efficacy and duration of CD19+ lymphocyte depletion in both groups. In the observation group, the median dose for complete B-cell depletion during the initial treatment was 0.1 g (interquartile range 0.1–0.5). In the standard group, all patients achieved B-cell depletion after the first 1 g RTX dose, and reassessment before the second 1 g dose at 2 weeks confirmed persistent depletion in all cases. During follow-up, B-cell reconstitution began at approximately 3 months after depletion in both groups, with no significant difference in B-lymphocyte counts at reconstitution between the observation group and the standard group (20.44 ± 29.52 vs. 20.98 ± 26.49, *t* = 0.07, *P* = 0.945).

### Cumulative RTX dosage and costs of immunosuppressive therapy

**FIGURE 4:**
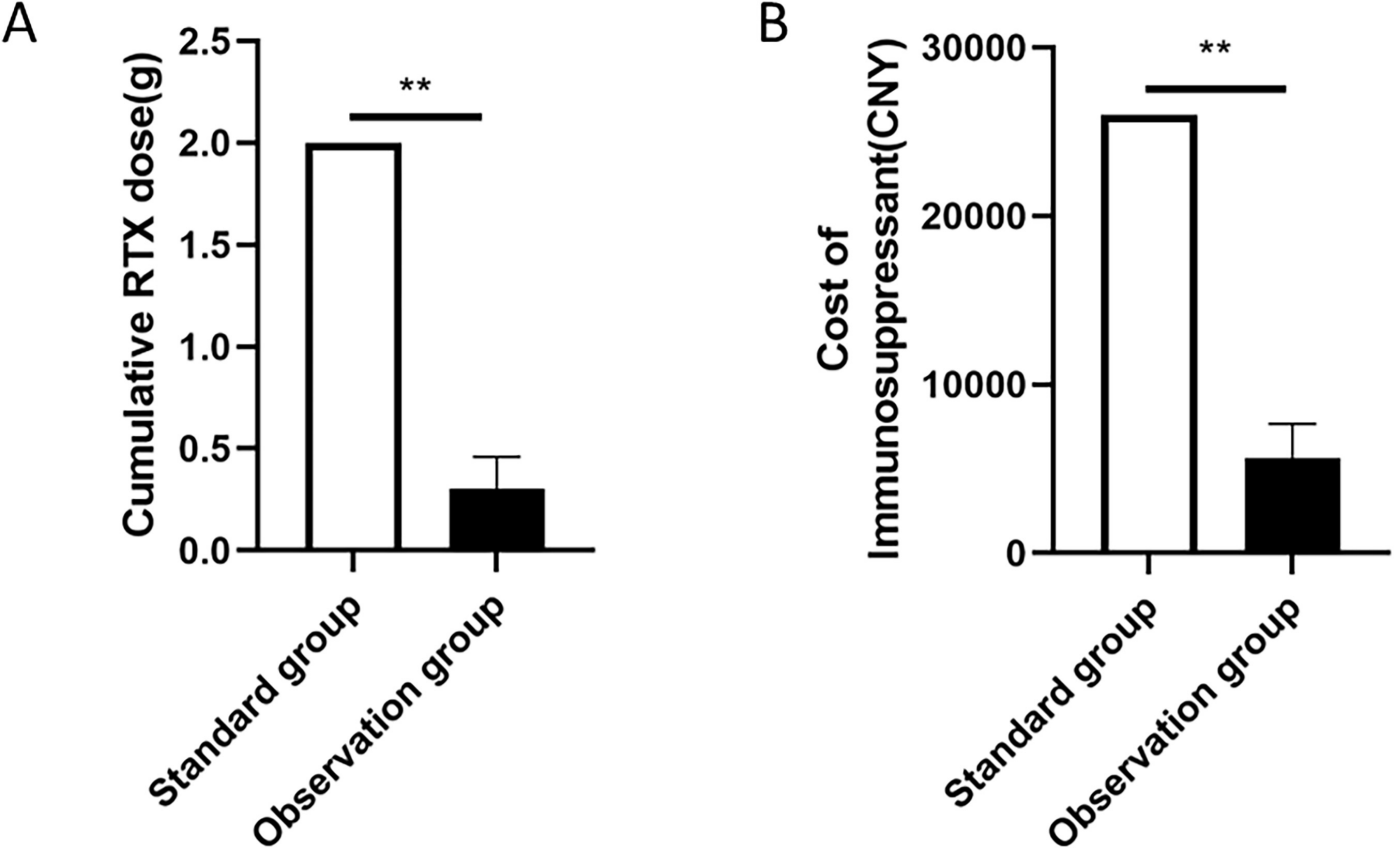
Comparison of RTX dosing **(A)** and immunosuppressive therapy costs **(B)** between the two groups. *P < 0.05, **P < 0.01, *P* < 0.05 indicates significant difference.

After 6 months, the treatment dose of RTX in the observation group was 0.3 ± 0.16 g, compared to 2 g in the standard group, with a statistically significant difference (*t* = 73.19, *P* = 0.000). The average cost of immunosuppressive therapy in the observation group was ¥5608.77 ± 2053.41 CNY, versus ¥26,000 CNY in the standard group. The observation group had significantly lower costs (*t* = 74.973, *P* = 0.000), resulting in an approximate saving of ¥20,391.23 CNY per person relative to the standard group (Figure 4).

### Incidence of adverse reactions

As of the 6-months treatment endpoint, no serious adverse events (SAEs) were reported in the observation group, whereas 4 SAEs were documented in the standard group, including 1 case of *Pneumocystis jirovecii* pneumonia, 1 case of autoimmune encephalitis, and 2 cases of severe pneumonia. Regarding non-serious adverse events (nSAEs), 12 events occurred in the observation group versus 18 in the standard group, primarily manifesting as infusion-related reactions (e.g., pruritus, rash, bronchospasm) and early post-administration events (e.g., herpesvirus reactivation, upper respiratory tract infection). The overall incidence of AEs in the observation group was significantly lower than that in the standard group (χ^2^ = 0.009, *P* = 0.013). No significant changes in renal function indicators from baseline were observed in either group, and there was no statistically significant difference between the two groups (*t* = −1.742, *P* = 0.086).

## Discussion

Our study demonstrates that in high-risk newly diagnosed or relapsed/refractory PMN patients, the combination regimen of B-cell-guided RTX plus low-dose TAC yields a therapeutic response comparable to the standard two-dose RTX regimen. There was no significant difference in the overall response rate at 6 months in the two groups. The average dose of RTX used was 0.3 g in the observation group, significantly lower than the 2 g in the standard group. Meanwhile, this new strategy carries a lower infection risk and yields an average saving of approximately ¥20,391 per patient in the cost of immunosuppressive therapy. The combination therapy of B-cell-guided RTX and low-dose TAC is expected to be a potential option for the treatment of PMN.

In recent years, PMN has been confirmed as an organ-specific autoimmune disease mediated by autoantibodies produced by B-cells against the antigen components of glomerular podocyte membranes (9, 12). Results from multiple randomized controlled trials, including GEMRITUX, MENTOR, STARMEN, and RI-CYCLO, show that RTX treatment targeting B-cells can achieve consistent remission rates comparable to conventional treatment regimens (21, 27–29). The KDIGO guideline and multiple expert consensuses recommend the standard-regimen RTX as the first-line treatment for PMN (30–32). Nevertheless, a high cumulative dose of RTX may induce a prolonged state of immunodeficiency, potentially doubling the susceptibility to infections. This heightened vulnerability is particularly concerning during the COVID-19 pandemic, as it may substantially increase the risk of severe disease through mechanisms such as delayed viral clearance, progression to critical illness, and diminished vaccine efficacy in patients with COVID-19 pneumonia (33, 34). Moreover, the substantial cost of high-dose RTX therapy imposes a significant financial burden on patients, prompting widespread clinical research aimed at refining treatment strategies–such as low-dose RTX regimens and combination therapies–to achieve better clinical outcomes and sustainability.

Bagchi et al. (35) demonstrated that a two-dose regimen of RTX at 500 mg per dose resulted in complete clearance of CD19+ B cells in 95.2% of patients with refractory PMN, with clinical remission achieved in 61.9% of patients. DEL-VECCHIO et al. (22) reported that rapid depletion of CD19+ B cells occurred following RTX infusion, independent of the administered dose. Ramachandran et al. (36) confirmed that a B-cell-guided RTX regimen could achieve efficacy comparable to two standard RTX regimens, noting that the clinical remission rate and duration in PMN patients were closely associated with CD19+ B cell levels. Furthermore, B-cell reconstitution was detectable prior to disease recurrence, indicating that the targeted depletion of CD19+ B cells may be a cost-effective strategy (37). However, some studies have indicated that while low-dose RTX can clear circulating B lymphocytes, the resulting disease remission is often suboptimal. In fact, 5%–28% of patients experience disease recurrence during the B-cell depletion phase (38). Calcineurin inhibitors (CNIs) effectively inhibit T cell activation and proliferation, modulating the Th17 immune response and facilitating disease remission (39–41). However, its application is constrained by the challenge of disease recurrence following dose reduction and the nephrotoxicity associated with long-term use. Based on the multi-target synergistic effects of RTX and CNIs in immunotherapy, this study compared the efficacy of a B-cell-guided RTX regimen combined with low-dose TAC against standard-dose RTX monotherapy. In our study, the overall remission rate for the combined regimen in PMN treatment at 6 months was comparable to that of standard RTX monotherapy. This outcome is attributed to the dual inhibitory effects of the combination therapy on both B lymphocytes and T lymphocytes, while also stabilizing the cytoskeletal proteins of podocytes (42), and inhibiting the “immune amplification loop” of complement activation (13), thereby providing ongoing protection to podocytes during the gradual recovery of B cells. In the initial round of the combined regimen, a median dose of 0.1 g (0.1, 0.5) of RTX was sufficient to achieve CD19+ B-cell depletion, with a mean total RTX dose of 0.3 g over 6 months. The regimen significantly reduced RTX exposure, thereby lowering the incidence of severe infections and overall treatment costs. And low-dose TAC effectively minimized CNI-associated nephrotoxicity while maintaining a favorable safety profile.

For patients with PMN, expert consensus suggests that when considering whether to re-administer RTX at 6 months post-treatment, the assessment should include B cell recovery status, anti-PLA2R antibody levels, and clinical remission status (31). In practice, however, persistent massive proteinuria without remission for 6 months carries a higher risk of renal function decline and thromboembolism. This situation also raises readmission rates and undermines patient adherence. Several studies report that monitoring peripheral blood CD19+ B cells and administering an additional RTX dose when their counts recover (CD19+ B cells ≥ 5 cells/mm^3^) effectively maintains remission and lowers the risk of relapse (25, 43). In this study, upon achieving B-cell depletion in the combined-medication group during the initial treatment phase, RTX was promptly readministered upon reappearance of peripheral blood CD19+ B cells to sustain depletion. Continuous monitoring showed that B-cell reconstitution began in both patient groups 3 months after treatment, suggesting that the duration of post-depletion maintenance in PMN patients may not depend strongly on the RTX dose. In some cases, B-cell reconstitution occurred alongside clinical remission. For example, one patient achieved rapid remission after a single 0.1 g dose of RTX. While his CD19+ B cell count increased at 3 months and normalized by 6 months, he maintained clinical remission for at least 18 months. We hypothesize that during B-cell reconstitution, transitional B cells emerge early while memory B cells recover later, potentially explaining why clinical relapse often follows B-cell repopulation. However, no clear temporal correlation has been established between the timing or extent of B-cell reconstitution and clinical outcomes, and the mechanisms underlying this dissociation remain unclear. Therefore, we propose that dynamic monitoring of B cell counts together with clinical remission status, followed by timely re-administration of RTX when indicated, may be more effective for achieving rapid disease control.

This study has several limitations. It is a single-center, retrospective cohort study based on HIS data from a tertiary-grade A hospital in China. The sample size is limited, with relatively few observed cases and a short follow-up period. Future high-quality randomized controlled trials are necessary to validate the long-term efficacy and safety of the B-cell-guided combination therapy of RTX plus low-dose TAC, as well as to monitor the potential development of anti-RTX antibodies during repeated low-dose RTX administration. Furthermore, differences in treatment cost structures, follow-up adherence, and other factors between the study setting and other countries or regions should be considered. Therefore, these findings should be applied within specific clinical contexts and patient populations, and should not be directly generalized to settings with differing healthcare policies or resource allocations.

## Conclusion

In conclusion, our study demonstrates that a B-cell–guided RTX combined with low-dose TAC regimen effectively induces clinical remission in both treatment-naive and relapsed/refractory high-risk PMN patients, while significantly reducing RTX exposure and lowering treatment costs. This strategy thus represents a safer and more cost-effective therapeutic option for high-risk PMN in resource-limited settings.

## Data Availability

The original contributions presented in this study are included in this article/supplementary material, further inquiries can be directed to the corresponding author.

## References

[1] Couser WG. Primary membranous nephropathy. *Clin J Am Soc Nephrol*. (2017) 12:983–97. 10.2215/CJN.11761116 28550082 PMC5460716

[2] Beck L Bomback AS Choi MJ Holzman LB Langford C Mariani LH et al. KDOQI US commentary on the 2012 KDIGO clinical practice guideline for glomerulonephritis. *Am J Kidney Dis*. (2013) 62:403–41. 10.1053/j.ajkd.2013.06.002 23871408

[3] Cattran DC Kim, Reich H Hladunewich M Kim SJ. Membranous nephropathy: quantifying remission duration on outcome. *J Am Soc Nephrol*. (2016) 28:995–1003. 10.1681/ASN.2015111262 27756808 PMC5328151

[4] McQuarrie EP Stirling CM Geddes CC. Idiopathic membranous nephropathy and nephrotic syndrome: outcome in the era of evidence-based therapy. *Nephrol Dial Transplant*. (2012) 27:235–42. 10.1093/ndt/gfr220 21558430

[5] Glassock RJ. Diagnosis and natural course of membranous nephropathy. *Semin Nephrol*. (2003) 23:324–32. 10.1016/s0270-9295(03)00049-4 12923720

[6] Kidney Disease: Improving Global Outcomes (Kdigo) Glomerular Diseases Work Group. KDIGO 2021 clinical practice guideline for the management of glomerular diseases. *Kidney Int.* (2021) 100:S1–276. 10.1016/j.kint.2021.05.021 34556256

[7] van den Brand JAJG Ruggenenti P Chianca A Hofstra JM Perna A Ruggiero B et al. Safety of rituximab compared with steroids and cyclophosphamide for idiopathic membranous nephropathy. *J Am Soc Nephrol*. (2017) 28:2729–37. 10.1681/ASN.2016091022 28487395 PMC5576929

[8] Beck LH Bonegio RG Lambeau G Beck DM Powell DW Cummins TD et al. M-type phospholipase A2 receptor as target antigen in idiopathic membranous nephropathy. *N Engl J Med*. (2009) 361:11–21. 10.1056/NEJMoa0810457 19571279 PMC2762083

[9] Miao H Zhang Y Yu X Zou L Zhao Y. Membranous nephropathy: systems biology-based novel mechanism and traditional Chinese medicine therapy. *Front Pharmacol*. (2022) 13:969930. 10.3389/fphar.2022.969930 36176440 PMC9513429

[10] Schlumberger W Hornig N Lange S Probst C Komorowski L Fechner K et al. Differential diagnosis of membranous nephropathy with autoantibodies to phospholipase A2 receptor 1. *Autoimmun Rev.* (2014) 13:108–13. 10.1016/j.autrev.2013.09.005 24075959

[11] Zhang P Huang W Zheng Q Tang J Dong Z Jiang Y et al. A novel insight into the role of PLA2R and THSD7A in membranous nephropathy. *J Immunol Res*. (2021) 2021:8163298. 10.1155/2021/8163298 34337081 PMC8298181

[12] Ruggenenti P Fervenza FC Remuzzi G. Treatment of membranous nephropathy: time for a paradigm shift. *Nat Rev Nephrol*. (2017) 13:563–79. 10.1038/nrneph.2017.92 28669992

[13] Fu H Tan Y. The complement system in membranous nephropathy: pathogenesis and targeted therapies. *Integr Med Nephrol Androl.* (2026) 13:e24–00059. 10.1097/imna-d-24-00059

[14] Wang YN Miao H Yu XY Guo Y Su W Liu F et al. Oxidative stress and inflammation are mediated via aryl hydrocarbon receptor signalling in idiopathic membranous nephropathy. *Free Radic Biol Med*. (2023) 207:89–106. 10.1016/j.freeradbiomed.2023.07.014 37451370

[15] Zhang W Chen J Yuan Y Luo J Zhou Z Wang G. PM2.5-induced oxidative stress upregulates PLA2R expression in the lung and is involved in the pathogenesis of membranous nephropathy through extracellular vesicles. *Front Pharmacol*. (2024) 15:1516111. 10.3389/fphar.2024.1516111 39744137 PMC11688400

[16] Miao H Wang YN Su W Zou L Zhuang SG Yu XY et al. Sirtuin 6 protects against podocyte injury by blocking the renin-angiotensin system by inhibiting the Wnt1/β-catenin pathway. *Acta Pharmacol Sin*. (2023) 45:137–49. 10.1038/s41401-023-01148-w 37640899 PMC10770168

[17] So BYF Yap DYH Chan TM. B cells in primary membranous nephropathy: escape from immune tolerance and implications for patient management. *Int J Mol Sci*. (2021) 22:13560. 10.3390/ijms222413560 34948358 PMC8708506

[18] Reff ME Carner K Chambers KS Chinn PC Leonard JE Raab R et al. Depletion of B cells in vivo by a chimeric mouse human monoclonal antibody to CD20. *Blood.* (1994) 83:435–45. 10.1182/blood.V83.2.435.435 7506951

[19] Dahan K Debiec H Plaisier E Cachanado M Rousseau A Wakselman L et al. Rituximab for severe membranous nephropathy: a 6-month trial with extended follow-up. *J Am Soc Nephrol*. (2017) 28:348–58. 10.1681/ASN.2016040449 27352623 PMC5198292

[20] Fervenza FC Appel GB Barbour SJ Rovin BH Lafayette RA Aslam N et al. Rituximab or cyclosporine in the treatment of membranous nephropathy. *N Engl J Med*. (2019) 381:36–46. 10.1056/NEJMoa1814427 31269364

[21] Seitz-Polski B Dahan K Debiec H Rousseau A Andreani M Zaghrini C et al. High-dose rituximab and early remission in PLA2R1-related membranous nephropathy. *Clin J Am Soc Nephrol*. (2019) 14:1173–82. 10.2215/CJN.11791018 31340979 PMC6682825

[22] Del Vecchio L Allinovi M Rocco P Brando B. Rituximab therapy for adults with nephrotic syndromes: standard schedules or B cell-targeted therapy? *J Clin Med*. (2021) 10:5847. 10.3390/jcm10245847 34945143 PMC8709396

[23] Shen X Jiang H Ying M Xie Z Li X Wang H et al. Calcineurin inhibitors cyclosporin A and tacrolimus protect against podocyte injury induced by puromycin aminonucleoside in rodent models. *Sci Rep*. (2016) 6:32087. 10.1038/srep32087 27580845 PMC5007516

[24] Chen X Jiao S Li S Li J Li P Song F et al. Combination of rituximab and low-dose tacrolimus in the treatment of refractory membranous nephropathy: a retrospective cohort study. *Balkan Med J*. (2023) 40:287–93. 10.4274/balkanmedj.galenos.2023.2022-9-7 37260416 PMC10339845

[25] Waldman M Beck LH Braun M Wilkins K Balow JE Austin HA. Membranous nephropathy: pilot study of a novel regimen combining cyclosporine and Rituximab. *Kidney Int Rep*. (2016) 1:73–84. 10.1016/j.ekir.2016.05.002 27942609 PMC5138549

[26] Waldman M Austin HA Balow JE. Rituximab or cyclosporine for membranous nephropathy. *N Engl J Med*. (2019) 381:1688. 10.1056/NEJMc1910393 31644855

[27] Fiorentino M Tondolo F Bruno F Infante B Grandaliano G Gesualdo L et al. Treatment with rituximab in idiopathic membranous nephropathy. *Clin Kidney J*. (2016) 9:788–93. 10.1093/ckj/sfw091 27994855 PMC5162414

[28] Gauckler P Shin JI Alberici F Audard V Bruchfeld A Busch M et al. Rituximab in membranous nephropathy. *Kidney Int Rep*. (2021) 6:881–93. 10.1016/j.ekir.2020.12.035 33912740 PMC8071613

[29] Scolari F Delbarba E Santoro D Gesualdo L Pani A Dallera N et al. Rituximab or cyclophosphamide in the treatment of membranous nephropathy: the RI-CYCLO randomized trial. *J Am Soc Nephrol*. (2021) 32:972–82. 10.1681/ASN.2020071091 33649098 PMC8017548

[30] Rovin BH Adler SG Barratt J Bridoux F Burdge KA Chan TM et al. Executive summary of the KDIGO 2021 guideline for the management of glomerular diseases. *Kidney Int*. (2021) 100:753–79. 10.1016/j.kint.2021.05.015 34556300

[31] Nephrology Expert Panel of the Peking University Health Science Center. [Expert consensus on the application of rituximab in the treatment of membranous nephropathy]. *Zhonghua Nei Ke Za Zhi.* (2022) 61:282–90. 10.3760/cma.j.cn112138-20210927-00660 35263969

[32] Chinese Society of Nephrology. Expert consensus on the use of rituximab in glomerulonephritis. *Chin J Nephrol.* (2022) 38:151–60. 10.3760/cma.j.cn441217-20210615-00023

[33] Jing Y Luo L Chen Y Westerberg LS Zhou P Xu Z et al. SARS-CoV-2 infection causes immunodeficiency in recovered patients by downregulating CD19 expression in B cells via enhancing B-cell metabolism. *Signal Transduct Target Ther*. (2021) 6:345. 10.1038/s41392-021-00749-3 34552055 PMC8456405

[34] Stefanski AL Rincon-Arevalo H Schrezenmeier E Karberg K Szelinski F Ritter J et al. B Cell Numbers predict humoral and cellular response upon SARS-CoV-2 vaccination among patients treated with rituximab. *Arthritis Rheumatol*. (2022) 74:934–47. 10.1002/art.42060 34962360 PMC9011692

[35] Bagchi S Subbiah AK Bhowmik D Mahajan S Yadav RK Kalaivani M et al. Low-dose Rituximab therapy in resistant idiopathic membranous nephropathy: single-center experience. *Clin Kidney J*. (2018) 11:337–41. 10.1093/ckj/sfx105 29942496 PMC6007352

[36] Ramachandran R Bharati J Rao I Kashif AW Nada R Minz R et al. Persistent CD-19 depletion by rituximab is cost-effective in maintaining remission in calcineurin-inhibitor dependent podocytopathy. *Nephrology*. (2019) 24:1241–7. 10.1111/nep.13554 30586217

[37] Ramachandran R Nayak S Kumar V Sethi J Minz R Kumar V et al. Rituximab in primary membranous nephropathy: a comparative study of three dosing regimens. *Nephrol Dial Transplant*. (2021) 36:1352–4. 10.1093/ndt/gfab037 33576822

[38] Sato M Kamei K Ogura M Ishikura K Ito S. Relapse of nephrotic syndrome during post-rituximab peripheral blood B-lymphocyte depletion. *Clin Exp Nephrol*. (2018) 22:110–6. 10.1007/s10157-017-1415-8 28434126

[39] Otsuka S Melis N Gaida MM Dutta D Weigert R Ashwell JD. Calcineurin inhibitors suppress acute graft-versus-host disease via NFAT-independent inhibition of T cell receptor signaling. *J Clin Invest*. (2021) 131:e147683. 10.1172/JCI147683 33822776 PMC8159692

[40] Li HY Zhang X Zhou T Zhong Z Zhong H. Efficacy and safety of cyclosporine a for patients with steroid-resistant nephrotic syndrome: a meta-analysis. *BMC Nephrol*. (2019) 20:384. 10.1186/s12882-019-1575-8 31646979 PMC6813125

[41] Christiansen D Mouhtouris E Hodgson R Sutton VR Trapani JA Ierino FL et al. Antigen-specific CD4+ CD25+ T cells induced by locally expressed ICOS-Ig: the role of Foxp3, Perforin, Granzyme B and IL-10 - an experimental study. *Transpl Int*. (2019) 32:1203–15. 10.1111/tri.13474 31225919

[42] Mo X Li J Liu Y Liao X Tan M Chen Y et al. Kidney podocyte-associated gene polymorphisms affect tacrolimus concentration in pediatric patients with refractory nephrotic syndrome. *Pharmacogenomics J*. (2020) 20:543–52. 10.1038/s41397-019-0141-x 31902946

[43] Ruggenenti P Cravedi P Chianca A Perna A Ruggiero B Gaspari F et al. Rituximab in idiopathic membranous nephropathy. *J Am Soc Nephrol*. (2012) 23:1416–25. 10.1681/ASN.2012020181 22822077 PMC3402291

